# Reaction of Rat Subcutaneous Connective Tissue to Resin Composites Polymerized with Different Light Curing Units and Different Lightening Methods

**DOI:** 10.1155/2012/156352

**Published:** 2012-06-14

**Authors:** Atiyeh Feiz, Farahnaz Arbabzadeh Zavareh, Seyed Mohammad Razavi, Hamid Badrian, Sepideh Dolatyar, Mansoureh Vajihi

**Affiliations:** ^1^Department of Operative Dentistry and Torabinejad Dental Research Center, School of Dentistry, Isfahan University of Medical Sciences, Isfahan 81746-73461, Iran; ^2^Department of Oral Pathology and Torabinejad Dental Research Center, School of Dentistry, Isfahan University of Medical Sciences, Isfahan, Iran; ^3^School of Dentistry, Isfahan University of Medical Sciences, Isfahan, Iran

## Abstract

The aim of the study was to determine and compare the reaction of rat subcutaneous connective tissue to resin composites polymerized with different lights curing and lightening methods. In this in vivo study, 20 mature Wister Albino rats were used. The composite discs, 4 mm in diameter and 2 mm thick, were cured by QTH or LED light curing units with 4 different lightning methods (full power QTH, full power LED, pulse LED, and ramp LED). Five resin composite discs were implanted in each rat, so that 4 of 5 discs for implantation of cured composite discs differently and central one as control without implantation. After sacrificing at 7, 14, 30, and 60 days the inflammatory grade, fibrosis, and necrosis were determined. Freedman and Cochran tests were used to analyze the data using SPSS software ver. 15. The results of the study showed significant differences in inflammation grade and fibrosis among control group and 4 experimental groups at day 14 (*P* < 0.05). In necrosis, there was no significant difference among 4 groups in different times (*P* > 0.05). In conclusion, neither the type of light curing units (LED or QTH) nor the lightening methods can affect the grade of inflammatory reaction.

## 1. Introduction

Resin composites have frequently been used as posterior restorative materials due to the demand for both aesthetic restorations and concerns over the adverse effects of mercury ingredient of amalgam. Adequate polymerization is a critical factor for increasing the physical properties and clinical performance of resin composite restorative materials. Residual uncured monomers or oligomers would cause cytotoxicity. Even in restorative materials which are fully set, substantial amounts of short-chain polymers remain unbound. Therefore there is probable elution of leachable toxic components toward the pulp. Also there is a relation between the amount of uncured reachable resin in the composite and the magnitude of the cytotoxic effect. In order to overcome the problem of inadequate polymerization, new methods, of curing, such as soft start and full power methods have been introduced [1]. 

Inadequate polymerization causes various problems such as inferior physical properties, solubility in the oral environment, and increased microleakage with resultant recurrent decay and pulpal irritation. The amounts of leachable residual monomers, on the other hand, could vary with the light source used for curing. Nowadays, various light curing units (LCUs) are available in dental practice, among which the halogen lamps are the most frequently used, although recently the light-emitting diode technology has been successfully proposed. Light-emitting diodes (LEDs), compared to Quartz Tungsten Halogen (QTH) lights, convert electricity into light more efficiently, produce less heat, and are more robust. Unlike the 30 to 50 hours life span of a conventional quartz tungsten halogen light bulb, light-emitting diodes last for thousands of hours [2].

One of the most important aspects of dental materials is their biocompatibility. Different methods for determining the biocompatibility of dental materials have been presented in the literature. Cytotoxicity tests conducted in vitro on the cell or tissue cultures and methods of implantation in subcutaneous connective tissue or bone in experimental animals are the basic ones [3].

 The presence of fibrous tissue and its disposition around implants of dental materials are indicative of tissue response. Therefore, the biocompatibility of a material is inversely related to the amount of fibrosis developed around it [4].

Researches conducting on biocompatibility of resin composites showed the accepted biocompatibility of these materials [3, 5]. Also, the other studies on cytotoxicity of resin composites cured with different curing units and different light-emitting methods have shown various outcomes. In a piece of research various modes of curing had no significant effect on cytotoxicity of tested composite materials [1]. In another study the type of light curing unit revealed significant effect on cytotoxicity of resin materials. LEDs light curing units resulted in the better cell survival than QTH [2]. Further, depending on the type of resin composites and curing methods, cytotoxicity significantly decreased [6].

Considering little information on biocompatibility of resin composites especially about the effect of the type of light curing unit and various curing methods, the aim of this study was to determine and to compare the subcutaneous connective tissue response in rats to resin composites cured by QTH light curing unit (full power method) and LED (ramp, pulse, full power methods).

## 2. Materials and Methods

In this experimental study, 20 mature male, 3-4-month Wister Albino rats weighting 150–200 grams were selected. Animals were cared according to the Public Health Service Policy on Human Care and Use of Laboratory Animals of the American Veterinary Medical Association Panel on Euthanasia, 2000 [3].

 The rats were deeply anesthetized. Four points with the maximum interspace were selected on the back of the rats. After shaving and disinfection, 4 incisions of 1 cm long and 15 mm deep were made using surgical scissors and hemostat forceps.

In order to make resin composite discs, dark rubbery washer-like molds (which had a cavity of 4 mm diameter and 2 mm depth) were used. A plastic strip was put on a glassy slab, the rubbery mold was placed on it, and composite Filtek-Z250 (3M ESPE, St. Paul, MN, USA, Table 1) was packed inside the mold's cavity with a condenser. The other side was covered with another plastic strip and pressed tightly to have even surface of resin composite after curing with LCU for 20 seconds.

The properties of light curing units and the methods applied for curing of resin composites were given in Table 2.

Prepared resin composite discs were placed in incised areas with the following order:

1. anterior right, resin composite cured with ramp mode of LED;
2. anterior left, resin composite cured with pulse mode of LED;
3. posterior right, resin composite cured with fast mode of LED;
4. posterior left, resin composite cured with fast mode of QTH.

In the control group, the center of dorsal skin of each rat was incised and sutured without implantation. The 3/0 silk sutures were used to close the skin.

**Figure 1:**
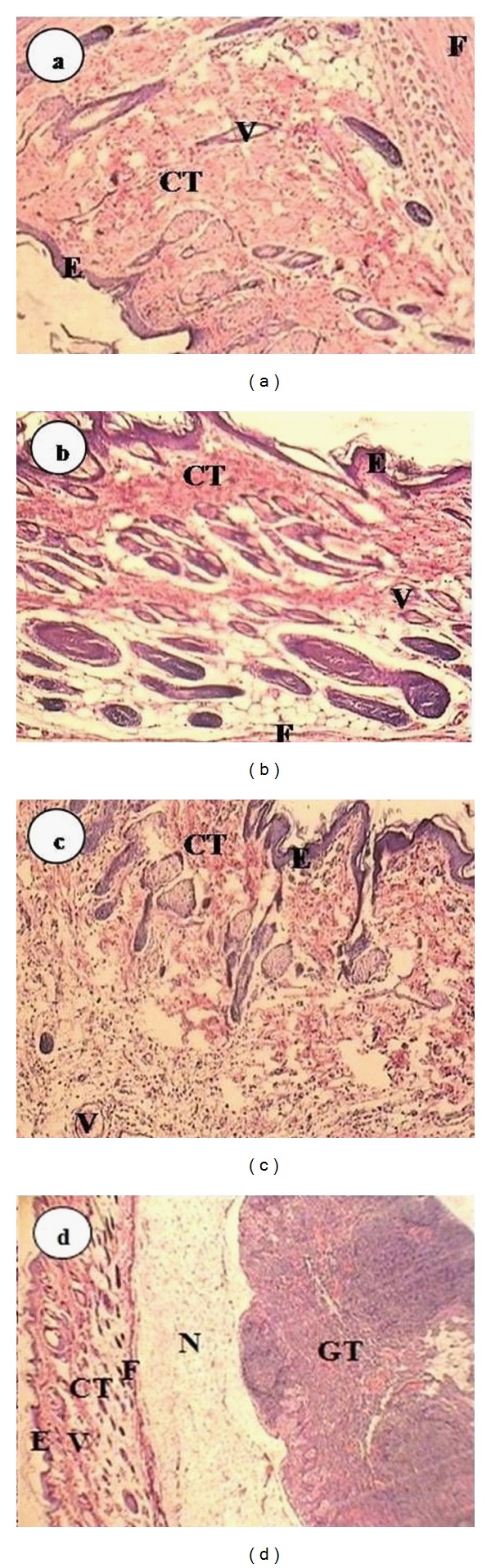
The photomicrographs of rat subcutaneous connective tissue reaction with ×100 magnification. (a) Grade 0 (b) Grade I (c) Grade II (d) Grade III. e: Epiderm, CT: connective tissue, CC: chronic inflammatory cell, GT: granulation tissue, F: fibrosis, N: necrosis, V: vessel.

After 7, 14, 30, and 60 days the implantation areas with 1 cm margin of subcutaneous connective tissue were collected and fixed in 10% formalin. Hematoxylin and Eosin stained slides were evaluated histopathologically under light microscope (Zeiss, Oberkochen, Germany). For counting the cells, magnification of ×40 and for evaluating the details, such as cells types, form of collagens and fibrous tissues magnification of ×100 were used. The amount of tissue inflammation and inflammation process was assessed on the basis of inflammatory cells next to the resin composites and also the whole number of inflammatory cells and it was graded with 4 number (grade 0 for no inflammatory response and grade III for severe response). The amount of tissue healing was also evaluated and scaled according to the number of fibroblastic cells and quality of fibrous tissue and collagen. Determining criteria for the inflammation grades are shown in Table 3. The photomicrographs of the inflammation grades are shown in Figure 1. To compare the grade of inflammation in different experimental groups in various time intervals the Friedman test was used and for statistical analysis of necrosis and fibrosis in different groups at various time intervals the Cochran test was employed (*α* = 0.05). 

## 3. Results

At 7 days, the maximum inflammatory response in all groups had grade I, except for control group (4 samples with grade 0). Grade II of inflammatory response was commonly observed at 14 days in all groups. There was a significant difference between control group and other groups (*P* < 0.05). Also, maximum response through the experimental period was observed at 14 days (grade II or III). The response decreased dramatically at 30 and 60 days. The degree of inflammation for each group was given in Table 4. At 7, 14, and 60 days more samples had thick fibrosis layer. At 14 days there were three LED pulse samples with narrow fibrosis, significantly different from control group (*P* < 0.05) (Table 5). 

 In the case of necrosis, no significant difference was observed among the groups at different observation periods (*P* > 0.05). At 14 days, at least one sample showed necrosis for composite implanted groups (Table 5).

## 4. Discussion

Nowadays, resin composites have been widely used in restorative dentistry despite having lots of problems. One of these problems is the polymerization shrinkage and consequent microleakage between tooth and restoration which decrease the longevity of restorations and even cause tooth necrosis. In order to overcome these problems various techniques are introduced for slow curing of the resin composites such as pulse and ramp techniques which are used in new light curing devices [2].

In comparison to curing with QTH, light-emitting diodes have advantages such as better curing, less heat, constant output over time without destruction, and longer life time. Furthermore, with the introduction of LEDs, it is claimed that the emission of blue light from LEDs provides an ideal spectra for curing of monomeric dental materials. Therefore, fewer toxic substances may leach in the environment. The quality of light curing devices could have an effect on biocompatibility of light curing materials [2]. Therefore in the present study the biocompatibility of resin composites cured with QTH and LED light curing devices was investigated, in which the LED was activated with different modes of light emitting.

Biocompatibility is one of the most important characteristics of dental materials. Various methods are used for assessing the biocompatibility of dental materials such as implanting them subcutaneously. It is shown that one of the best ways for assessing the local effects of dental materials is a subcutaneous implantation in laboratory animals. Toxic and inflammatory reactions to implanted dental materials are specific reactions which are observed in all connective tissues [3].

In histopathological studies, the size and shape of implanted materials have effects on tissue reactions [3]. While in some studies the specimens were directly implanted into subcutaneous connective tissues [4], in the others they were placed in tubes and then implanted [3, 5]. In this study 2 mm thick resin composite discs were used to mimic clinical conditions.

In the present study in order to assess the short-term and long-term reactions, the evaluations were made after 7, 14, 30, and 60 days according to specification of American National Standards Institute [7].

Different light curing devices and lightening modes may affect the release of resin monomers which have a potential effect on biocompatibility and cytotoxicity of dental materials [2, 8–10]. Photopolymerization of resinous materials would create a solid phase hence significantly decreasing the amount of free monomer and substantially reducing the potential for harmful stimuli. Complete polymerization may diminish all the irritants but cured resins never completely polymerized and they degraded over time. Releasing unpolymerized monomers such as triethylene glycol dimethacrylate (TEGDMA), 2-hydroxy-ethyl-methacrylate (HEMA), or bisphenol A glycerolate dimethacrylate (BISGMA) could cause adverse reactions in cell cultures [11–14]. TEGDMA and bis-GMA are releasable monomers from Filtek-Z250.

 In the present study, mild or moderate inflammatory response to resin composites was observed in the first week, and then progressing to moderate and severe reactions in the second week. This could be the result of unreacted free monomers which were released gradually and caused more inflammatory reactions in the second week. Also, it is speculated that rat immune system might be delayed to react against foreign bodies. This would be a reason for moderate or severe response to the light-cured resin composites. After one or two months the inflammatory responses declined to moderate and low reactions due to elimination of the superficial free monomers by immune cells. Formation of fibrosis around the resin composite was explaining the well tissue tolerance [3]. The findings of the present study were in agreement with Ozbas et al. study [3], except for the maximum inflammatory reaction. In Ozbas study maximum inflammatory reaction was observed in day 7, but in the present study it was observed in day 14. This difference could be correlated to the type and intensity of LCU, degree of polymerization, type of resin composite, size and shape of tested materials, and the implantation methods.

In this study, the inflammatory reactions to cured resin composite with different devices (QTH and LED) and various lightening methods had not significant differences. The findings of the present study are the same as the findings of Nalçaei et al. [1] and Ergun et al. [10, 15] in which they assessed the cytotoxicity of resin composites polymerized with different curing devices and various lightening methods.

It is well known that the curing degree of resin composite is related to energy density and exposure time. If the polymerization parameters are not complete, unsatisfactory curing would happen, leading to the release of the much more free monomers from the resin composites. These free monomers are toxic and could reach the pulp tissue through dentinal tubules [6].

In conclusion, according to the limitation of this study, the type of light curing unit (QTH, LED) and method of resin composite curing (ramp, pulse, full power) had no effect on inflammatory reaction to the resin composites. The intensity of inflammatory reaction was low to moderate in the first week, moderate to severe in the second week and then decreased to low to moderate after 1 or 2 months. Formation of fibrous connective tissues showed well tissue tolerance to the resin composites.

Further studies with more focus on the effect of different light curing units such as plasma arch and laser with various curing modes and different methods for testing biocompatibility are suggested.

## Figures and Tables

**Table 1:** Ingredients of composite Filtek-Z250 (3M, ESPE, St. Paul, MN, USA).

Ingredients	% by Wt
Silane treated ceramic	75–85
Bisphenol a polyethylene glycol diether dimethacrylate (BISEMA6)	1–10
Diurethane dimethacrylate (UDMA)	1–10
Bisphenol-A diglycidyl-ether-dimethacrylate (bis-GMA)	1–10
Triethyleneglycol-dimethacrylate (TEGDMA)	<5

**Table 2:** Types of light curing units, their intensity and methods of curing.

Type of LCU	Brand	Manufacturer	Curing mode	Light intensity output	Curing methods
QTH	Coltolux50	Coltene/Whaledent, USA	Full power	620	Continuous energy output for 20 sec.
LED	LED Turbo	Apoza Enterprise, Taiwan	Full power	600	Continuous energy output for 20 sec.
LED	LED Turbo	Apoza Enterprise, Taiwan	Ramp	+600	Automatically increase to full energy within 2 sec. + 18 sec. full energy
LED	LED Turbo	Apoza Enterprise, Taiwan	Pulse	600, 0, 600, 0	Full energy for 0.8 sec. with 0.2 sec. interval

**Table 3:** Criteria for Scaling of inflammation degrees.

No inflammation (Grade 0)	Mild inflammation (Grade I)	Moderate inflammation (Grade II)	Severe inflammation (Grade III)
No inflammatory cell	Presence of macrophages and/or plasma cells	Presence of macrophages and plasma cells	High density of inflammatory cells
Fibroblasts > 30	Inflammatory cells < 30	Accumulations of granulocytes and/or lymphocytes	Inflammatory cells ≥ 60
		30 ≤ Inflammatory cells < 60	
Mature fibrous tissue with many collagen fibers	10 < fibroblasts < 30	5 < Fibroblasts < 9	Local necrosis areas
	Immature fibrous tissue with few collagen fibers		1 < Fibroblasts < 4

**Table 4:** Inflammation degree produced by light curing units in different modes of lightening.

	Day 7				Day 14				Day 30				Day 60			
	Inflammation degree				Inflammation degree				Inflammation degree				Inflammation degree			
LCU (mode of lightening)	Gr. 0	Gr. I	Gr. II	Gr. III	Gr. 0	Gr. I	Gr. II	Gr. III	Gr. 0	Gr. I	Gr. II	Gr. III	Gr. 0	Gr. I	Gr. II	Gr. III

QTH (full power)	1	3	1	0	0	1	4	0	1	4	0	0	1	2	2	0
LED (full power)	2	2	1	0	0	0	3	2	3	1	0	1	3	2	0	0
LED (ramp)	1	3	1	0	0	2	1	2	2	3	0	0	2	3	0	0
LED (pulse)	1	4	0	0	0	1	3	1	1	4	0	0	2	3	0	0
Control	4	1	0	0	1	4	0	0	4	1	0	0	3	1	0	0

Gr: Grade.

**Table 5:** Necrosis and fibrosis produced by light curing units in different modes of lightening.

	Day 7				Day 14				Day 30				Day 60			
	Necrosis		Fibrosis		Necrosis		Fibrosis		Necrosis		Fibrosis		Necrosis		Fibrosis	
LCU (mode of lightening)	Yes	No	T	N	Yes	No	T	N	Yes	No	T	N	Yes	No	T	N

QTH (full power)	0	5	5	0	1	4	5	0	0	5	4	1	0	5	5	0
LED (full power)	1	4	4	1	1	4	5	0	1	4	3	2	1	4	5	0
LED (ramp)	1	4	5	0	1	4	5	0	0	5	4	1	0	5	4	1
LED (pulse)	0	5	4	1	2	3	2	3	0	5	3	2	0	5	4	1
Control	0	5	5	0	0	5	5	0	0	5	5	0	0	5	5	0

Presence or absence of necrosis was determined as (Yes) or (No).

The thickness of fibrosis was shown as thick (T) or narrow (N).
